# Application of Real-World Data to External Control Groups in Oncology Clinical Trial Drug Development

**DOI:** 10.3389/fonc.2021.695936

**Published:** 2022-01-06

**Authors:** Timothy A. Yap, Ira Jacobs, Elodie Baumfeld Andre, Lauren J. Lee, Darrin Beaupre, Laurent Azoulay

**Affiliations:** ^1^ Department of Investigational Cancer Therapeutics (Phase I Program), Division of Cancer Medicine, the University of Texas MD Anderson Cancer Center, Houston, TX, United States; ^2^ Pfizer Inc., New York, NY, United States; ^3^ Centre for Clinical Epidemiology Lady Davis Institute, Jewish General Hospital, Montreal, QC, Canada; ^4^ Department of Epidemiology, Biostatistics, and Occupational Health and Gerald Bronfman Department of Oncology, McGill University, Montreal, QC, Canada

**Keywords:** cancer, clinical trial, design, drug development, external control group, oncology, RCT, real-world data

## Abstract

Randomized controlled trials (RCTs) that assess overall survival are considered the “gold standard” when evaluating the efficacy and safety of a new oncology intervention. However, single-arm trials that use surrogate endpoints (e.g., objective response rate or duration of response) to evaluate clinical benefit have become the basis for accelerated or breakthrough regulatory approval of precision oncology drugs for cases where the target and research populations are relatively small. Interpretation of efficacy in single-arm trials can be challenging because such studies lack a standard-of-care comparator arm. Although an external control group can be based on data from other clinical trials, using an external control group based on data collected outside of a trial may not only offer an alternative to both RCTs and uncontrolled single-arm trials, but it may also help improve decision-making by study sponsors or regulatory authorities. Hence, leveraging real-world data (RWD) to construct external control arms in clinical trials that investigate the efficacy and safety of drug interventions in oncology has become a topic of interest. Herein, we review the benefits and challenges associated with the use of RWD to construct external control groups, and the relevance of RWD to early oncology drug development.

## Introduction

Randomized controlled trials (RCTs) are considered the most reliable study method for providing data on the effects of a therapeutic intervention (1). The randomization limits bias by controlling for both known and unknown confounding factors. In oncology, RCTs assessing overall survival (OS) are considered the “gold standard” when evaluating the efficacy and safety of a new intervention (2). However, many RCTs do not measure OS as a primary endpoint, and limitations with study design, analysis, and conduct (e.g., deviations from intended interventions, missing outcome data, and measurement of the outcome) can place RCTs at high risk of bias (3). Even well-designed RCTs have drawbacks. For example, such trials require large numbers of patients, and it is often not feasible or ethical to recruit patients to control groups in rare diseases or in diseases where no effective standard-of-care treatments are available (4–6). Owing to the possibility of being randomized to receive a control treatment, patients may be less likely to enroll in an RCT than an uncontrolled, single-arm trial where all participants receive the investigational treatment (1). In addition, analysis of OS in an RCT requires prolonged follow-up of patients (7) and frequent patient crossover from the control arm to the active treatment arm, which is sometimes required for ethical reasons (8), could be perceived as misclassification of exposure in the context of an intent-to-treat analysis of OS (2).

Although not relevant to early phase trials, the utility of single-arm trials is noted for Food and Drug Administration (FDA) accelerated or breakthrough regulatory approval of a number of precision oncology drugs, for which the target patient populations are typically small (9). However, small, short-term, single-arm studies are inadequate in size or duration to acquire mature survival data. Rather, such studies use surrogate endpoints, such as objective response rate or duration of response, to measure clinical benefit. In addition, the lack of a standard-of-care comparator arm in single-arm trials can make interpretation of efficacy and safety challenging (10). Without an internal control group (i.e., a group comprising patients from the same population assigned to a different treatment) (11), assessments are performed by making indirect comparisons (7), which may be suboptimal. Over-optimistic interpretation of the results of single-arm studies can result in failures in subsequent large statistically-powered phase III trials (1).

**Figure 1:**
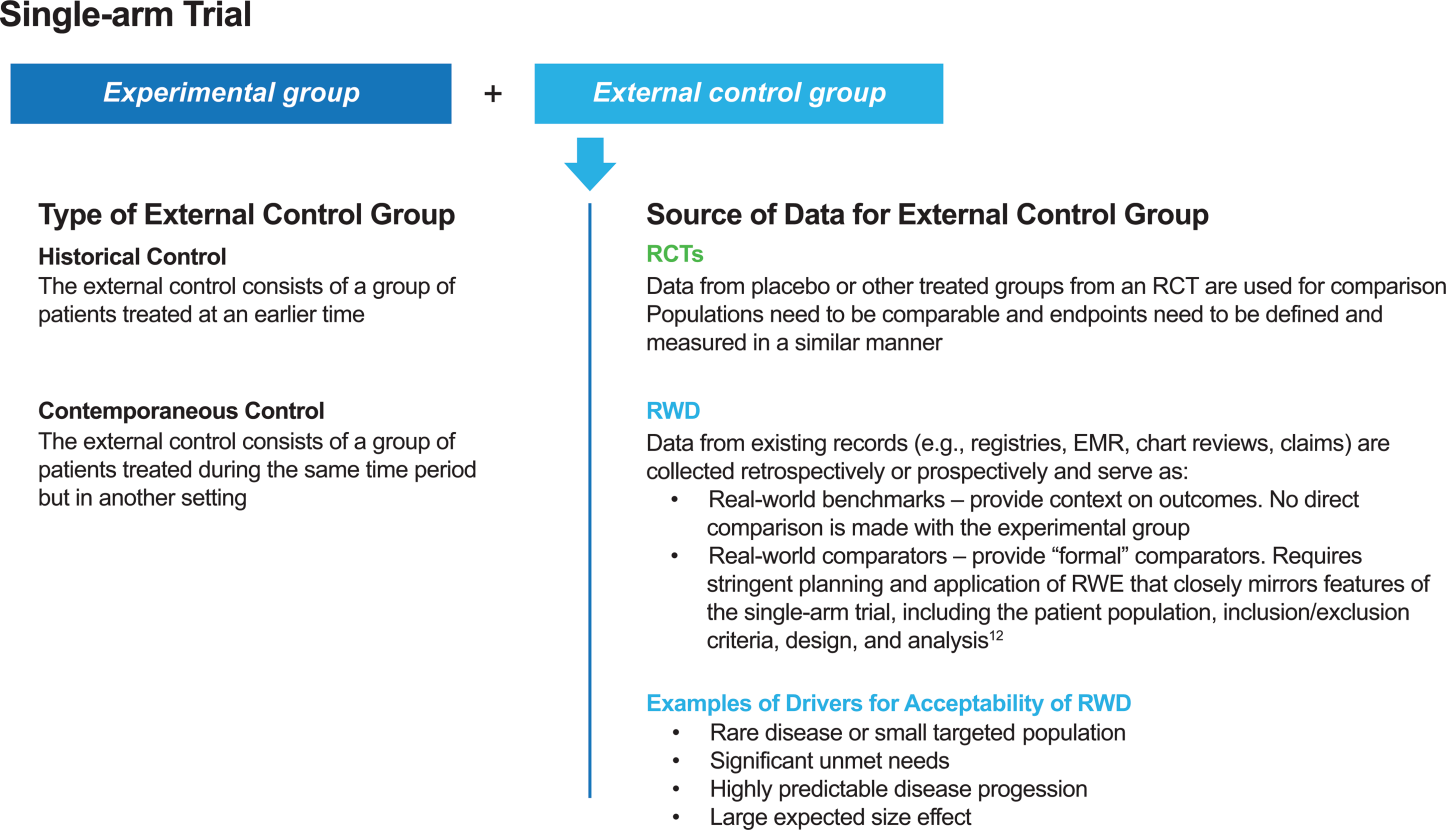
Types of external control groups and sources of data for external control groups. EMR, electronic medical record; RCT, randomized controlled trial; RWD, real-world data; RWE, real-world evidence.

Discussions about the limitations of RCTs have been ongoing for many years, and the use of a comparator based on data collected outside of a study — referred to as an external control or synthetic control group — could offer a compromise between uncontrolled trials and RCTs to address key research questions in a certain context (1, 12). An external control group could consist of patients treated at an earlier time (sometimes referred to as an historical control) or patients treated during the same period of time but in a different setting (sometimes referred to as a contemporaneous control) (11–13) (**Figure 1**). Such external controls may be based on clinical trial data or real-world data (RWD) (12, 13). The choice of data source depends on the research question. For example, clinical trial data would be an appropriate source for constructing external controls when the endpoints of interest need to be defined and measured in a similar manner. However, RWD may be better suited for constructing external controls when experience from clinical trials with a disease or disease subtype is limited (5).

The potential of external control groups that leverage RWD is currently of particular interest. Notably, at a workshop at the National Academy of Sciences, Engineering and Medicine in 2017 (14), the director of the FDA Center for Drug Evaluation and Research was quoted as saying the clinical trials system is “broken,” and new ways to collect and utilize patient data are needed. The FDA also added that there has been “very little historical use of real-world experience in drug regulatory decisions about effectiveness”, highlighting a gap for the utility of such data in drug development (14). This review considers the use of external controls as a comparator arm to clinical trials, and we consider the relevance and limitations of RWD-based external controls in early oncology drug development.

## The Evolving Landscape of Real-World Data

There is currently significant interest in leveraging RWD for clinical evidence generation in oncology (15). RWD include information obtained from electronic health records (EHRs), medical claims and billing databases, registries, patients’ records from in-home-use settings, and from other sources that can reveal health status (e.g., mobile devices) (16). The adoption of EHRs in recent years has been a major contributor to the emergence of RWD as an important source of clinical evidence (4, 15). In conjunction with improvements in data analytics, EHRs have not only made real-world evidence (RWE) generation more feasible and less costly, but they have also led to the creation and growth of companies specializing in the use of EHR data to support pharmaceutical product discovery and regulatory approvals (17).

**Figure 2:**
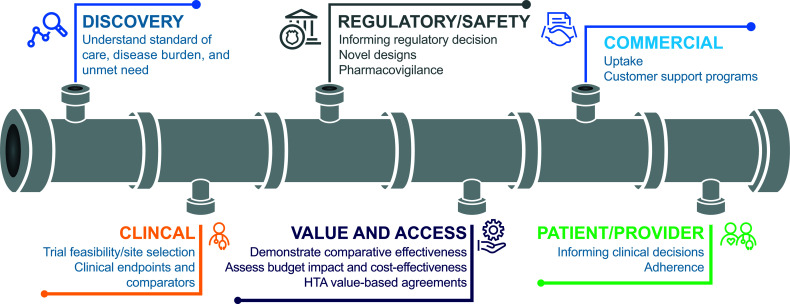
Uses for RWD throughout the drug development cycle (18). HTA, health technology assessment.

RWD could be utilized in numerous ways throughout the drug development cycle (**Figure 2**) (15, 17–20). During early development, in drug discovery, RWD may be used to characterize disease progression and the associated unmet need. For example, patient and environmental factors that influence the risk of cancer or the progression of cancer into a more advanced stage may be identified through the retrospective extraction of data from EHRs (15). RWE from this type of retrospective study may be used to inform clinical trial design and execution (15). In addition, RWD can provide a basis for power calculations, a prior for a Bayesian statistical analysis, or it can provide an external control group and guide enrichment. Furthermore, RWD may support the selection of representative patient populations and could be used to facilitate the discovery and validation of biomarkers.

In a post-approval setting, EHR-generated RWD would allow access to large, clinically relevant patient populations that could include patients who may be excluded from RCTs, such as older patients or patients with hepatic impairment (9). This use of RWD can broaden the understanding of treatment effects on patients who are routinely underrepresented in clinical trials (17). In addition, RWD can be used to address post-marketing questions about safety, such as long-term toxicities and complications that generally are not captured in clinical trials (17). RWD collected from EHRs could also provide information on patient characteristics (e.g., biomarker prevalence) and treatments (e.g., different lines and sequences of therapies, and standard-of-care treatments not selected as comparators in RCTs) in a large patient population. Furthermore, EHRs provide opportunities to measure survival without being impacted by crossover and to perform long-term follow-up (9).

Studies with an external control group based on RWD are not intended to replace RCTs or single-arm trials. However, when such studies are well conducted — and well designed to balance patient characteristics across study arms — RWD can be highly informative. For example, although not in an oncology indication, Patorno et al. (21) demonstrated that preliminary results of the CAROLINA study comparing cardiovascular safety of linagliptin and glimepiride in patients with type 2 diabetes at increased cardiovascular risk could be replicated using RWD collected from US claims data sets. In their study, the authors identified the patient population by adapting eligibility criteria from the CAROLINA study and by using propensity score matching to control for >120 potential confounding variables (21). In addition, the authors performed multiple prespecified validity checks before analyzing the primary outcome to confirm the study’s ability to replicate known causal associations for selected control outcomes (21).

As most EHRs were designed primarily to support billing and practice management rather than clinical research, there are many challenges associated with retrieving information for research purposes. In an ideal setting, to reliably and consistently use RWD for benchmarking in clinical research, it would be necessary that healthcare practitioners systemically record clinical observations rather than only recording information relevant for reimbursement. To address this issue, technological advances (e.g., natural language processing and data abstraction) provide an opportunity to generate potential datasets with the information collected in EHRs (15). As it is done in the context of observational studies, software can be used to extract structured data (e.g., cancer diagnosis codes), whereas abstraction of unstructured data (e.g., tumor histology from pathology reports) can be used to supplement structured data elements (18).

Aside from the quality limitations of using unstructured data (e.g., inaccurate, incomplete, or unclear data entries) (9), there are also technical complexities that must be considered when handling missing data or covariate information and when collecting RWD from selective sites to avoid potential for bias. Furthermore, RWD may be incompatible with other data sets or other platforms for data exchange. For instance, tumor assessments in clinical trials follow the Response Evaluation Criteria in Solid Tumors, whereas the same assessment follows other criteria in clinical practice (9); hence, comparisons between RWD-based endpoints and clinical trial outcomes may be limited. In addition, complete standardization of RWD may not be possible because methodology considerations, such as data collection and analysis, vary significantly between studies (9). Other challenges of using RWD collected from EHRs include difficulties with funding and resources for establishing, maintaining, and using EHRs; exclusion of data from countries and settings without EHR systems; data ownership and patient privacy; consent to secondary use of data; and acceptance of data and methodologies (9).

## The Role of RWD in Regulatory Decisions

In the past, the FDA has primarily used RWE for regulatory decisions about drug safety in the post-marketing setting. For example, the FDA has relied on real-world post-marketing safety surveillance to provide information on adverse events that may occur with low frequency or after a long follow-up period (22). Less frequently, the FDA has used RWE for regulatory decisions related to drug efficacy (22). One example is the approval of blinatumomab (Blincyto) by the FDA for relapsed/refractory acute lymphoblastic leukemia in 2014 (described in case 2 below) (23). In this case, the single-arm trial of blinatumomab was supported by historical control group data that were extracted from chart review of patients from US and European study sites who were treated with standard-of-care salvage chemotherapy (23, 24).

More examples of using RWE of efficacy to support FDA approvals of new drug indications in oncology were recently reviewed by Feinberg et al., 2020 (22). In one case, the FDA granted lutetium Lu 177 dotatate (a radiolabeled somatostatin analog) orphan drug designation for the treatment of somatostatin receptor-positive (SSTR-positive) gastroenteropancreatic neuroendocrine tumors on the basis of data from a randomized, open-label, active controlled trial (NETTER-1) and supporting RWE from a retrospective, investigator-sponsored, open-label, single-arm, expanded access study (ERASMUS) of patients with SSTR-positive neuroendocring tumors (22, 25).

However, in some cases, RWD may not be successful in supporting regulatory approval. One example is for selinexor, a small molecule inhibitor of the nuclear export protein, exportin 1, which was granted orphan drug designation by the FDA for the treatment of patients with relapsed refractory multiple myeloma (22, 26). The initial new drug application submission for selinexor included data from an open-label, single-arm trial (STORM) and a retrospective observational study that used EHR data from the Flatiron database (22, 26). However, the FDA identified methodological issues with the EHR data and results from the observational study were deemed inadequate to support regulatory decision making (22, 26). Therefore, EHR data were not considered in the approval decision. Rather, approval was granted on the basis of data from the STORM trial, data from an ongoing phase 3, randomized trial (BOSTON) and a post-marketing requirement to submit the final data from the BOSTON trial (22, 26).

The FDA has created a framework for evaluating the potential for RWE in supporting new indications for approved drugs or to support or satisfy post-approval study requirements (16). Other regulatory agencies, such as the European Medicines Agency (EMA) and Japanese Pharmaceuticals Medical Devices Agency (PMDA), have also expressed interest in using RWD to support regulatory decisions or postmarketing obligations (27–29).

## Limitations of RWD

RWD can provide valuable and complementary information to RCTs. However, there are some limitations of using RWD. For example, as RWD sources (e.g., EHRs or claims data) are not designed for clinical research there is the risk that potentially unobserved factors (e.g., physician opinion or patient request) have influenced a physician’s decided course of treatment, which prevents a direct comparison of outcomes between treatment arms or to RCT findings (30). In addition, patients in routine clinical practice may not be monitored as closely as patients in clinical trials, which may lead to reporting of lower rates of adverse events. It is also possible that minor adverse events may not be captured or recorded in the context of more serious diagnoses (30). In this way, RWD may underestimate safety outcomes. Other examples of limitations to RWD include a lack of pre-trial registration, which provides methodological transparency and serves to prevent multiple hypothesis testing; the inability to compare RWD before an experimental treatment is approved; and challenges in ensuring accuracy and completeness of data and loss of follow-up (30).

## Methodological Considerations in Incorporating External Control Data Into a Clinical Trial

### Selecting an External Data Source: Clinical Trials or RWD?

Compared with RWD, clinical trial data are collected more meticulously, with standardized definitions and protocols. External controls based on historical clinical trial data are most appropriate for well-studied conditions for which the standard of care has not changed much over time (e.g., small-cell lung cancer) (5). However, leveraging RWD as a source for external controls has some benefits (1, 5). For example, RWD is generated during routine clinical care, and hence may be available at a larger scale. In addition, RWD is more likely to be readily available for rare diseases or subgroups that may not have been studied extensively in clinical trials (1, 5). Furthermore, RWD may be important in settings where patients in RCT control groups frequently switch across to the investigational treatment arm. In some cases, patients are switched inadvertently, while in other cases patients are allowed to cross over from the control group to the investigational treatment arm because of ethical issues, such as in the study of panitumumab in metastatic colorectal cancer (8, 31).

A potential disadvantage in using RWD is that it is subject to both information and confounding biases. For example, patient characteristics may systematically differ between patients in the external control group and patients in the experimental treatment group (confounding bias) or data may be misclassified (information bias) (32). In addition, outcomes of interest (e.g., survival) could differ between patients in an external control group based on historical RWD and patients in the experimental treatment group as a function of time (time-related bias) (33). To this end, authors from Flatiron Health have published a checklist for ensuring “regulatory-grade” RWE (**Table 1**), stating that it should be high quality, complete, transparent, generalizable, timely, and scalable (18). Nevertheless, issues of confounding will persist even with the best available data. However, these potential biases can be minimized through study design and/or statistical techniques (34, 35).

**Table 1:** A checklist for ensuring regulatory-grade RWE (19).

Checklist Item	Explanation
High quality	“The provenance of each data point must be clear, traceable, and auditable. Data quality must be systematically measured with predetermined frameworks (e.g., interrater reliability) and against benchmarks (e.g., stage distribution in SEER).”
Complete	“Completeness requires predefined rules for abstraction of structured and unstructured data, data harmonization, and quality monitoring. Completeness needs to be benchmarked to appropriate gold standards (e.g., National Death Index for date of death).”
Transparent	“Transparent study designs and analysis plans are critical for robust RWE. In particular, the specific aims and cohort selection criteria need to be precisely defined. Study design considerations include retrospective vs. prospective data collection, the need for matching or propensity scores to facilitate comparisons, and endpoint validation.”
Generalizable	“RWE is often based on a broad range of patients, which can translate into better generalizability. Potential biases (e.g., geographic representation) must be identified and reported to allow for appropriate statistical adjustments and clinical interpretations.”
Timely	“RWE reflects daily clinical decisions. Thus, reliable RWE needs to be recent and timely. Details about the timepoint that the data analysis represents must be reported (e.g., time period, last update, number of potential candidates, etc.).”
Scalable	“Data challenges become exponentially more complicated as the number of patients and variables increase. Therefore, scaling requires 1) a balance between high touch and automation; 2) a modular data model that can be used in multiple contexts and facilitates model evolution (e.g., frequency of intravenous regimens); and 3) unambiguous variable definitions, particularly for endpoints.”

RWE, real-world evidence; SEER, Surveillance, Epidemiology and End Results. “Harnessing the Power of Real‐World Evidence (RWE): A Checklist to Ensure Regulatory‐Grade Data Quality” by Miksad RA et al. is licensed under CC BY-NC 4.0.

### Ensuring Comparability Between the Experimental Treatment and External Control Groups

A major limitation of externally controlled trials is the potential for confounding bias, i.e., a difficulty in establishing comparability between the treatment and control groups (11). Apart from study treatment, the groups could differ from one another in a variety of factors, which could affect outcome. Such factors could include important unrecognized and unmeasured prognostic variables. As noted by Gray et al., to ensure an internally valid comparison, the external and internal populations should ideally be exchangeable with each other with respect to: eligibility criteria, patient characteristics, mode of treatment (e.g., surgery, chemotherapy, radiation therapy), outcome measure, time period, and setting (6). However, as external data are typically collected using different methods and from different sites than where the trial is being conducted (36), such criteria are unlikely to be met. This is particularly true when using RWD, which are accrued without the same level of uniformity as clinical trial data and are not based on a common protocol (36).

Appropriately accounting for differences between the internal and external populations is a key consideration in avoiding bias and inappropriate conclusions from the comparison. A detailed examination of the statistical methods for using external comparators, including propensity score methods and Bayesian methods, is outside the scope of this paper, and interested readers are directed to a recent review by Lim et al. (37). In short, propensity score methods are important in eliminating or reducing potential bias in estimated effects observed in non-randomized comparisons, and have been applied for externally controlled trials (10, 37). As described by Schmidli et al. (38), eligibility criteria of the single-arm trial are used to select a subset of individual patient data from the external data in order to reduce the difference between the datasets. Then, baseline information is used to estimate the propensity to be eligible for the new trial, *via* logistic regression or machine learning techniques, and finally, patients are matched using the propensity score such that they are comparable between the two groups (38).

The main assumption with this technique is that all differences between the external and internal populations are explained by baseline covariates (38). The propensity score may also be used in other ways, such as through propensity score weighting (10, 38). This approach of propensity score weighting would allow for the use of data from all available patients meeting the trial eligibility criteria and, thereby, maximize the statistical power of the study. However, propensity score methods have some limitations. For example, the use of propensity score methods can only correct for known baseline confounders; however, there could be unknown confounders that may not be accounted for. In addition, propensity score matching could change the population being studied through exclusion of some subset of patients who fail to achieve a proper match in the other “arm” of the analysis (30). As a result, propensity score matching “may provide causal estimates about the effect of the intervention on a different population than the study originally sought to investigate” (30).

The selection of patients for external control groups would need to consider the severity of disease. Most patients in phase I studies have advanced cancers with limited or no treatment options. In this case, it may be appropriate to select patients who have previously received all approved agents when generating an external control group from RWD to ensure comparability between the control and treatment groups. In addition, other study design choices, such as comparator group(s), temporality, and method of propensity score adjustment, should be considered when selecting external control groups based on RWD (39).

## Examples of RWD Used as an External Control in Oncology

### Case 1: Proof-Of-Concept Study of an EHR-Based External Control Group in Patients With Anaplastic Lymphoma Kinase-Positive Non-Small Cell Lung Cancer

Davies et al. (40) aimed to compare the OS of patients with anaplastic lymphoma kinase-positive (*ALK+*) NSCLC treated with alectinib vs. those treated with ceritinib following crizotinib treatment failure. Without comparative evidence from RCTs, the authors derived an alectinib treatment arm by pooling data from two single-arm phase II studies and constructed an external ceritinib control group using RWD. The authors noted that externally controlled studies can be susceptible to systematic variation or bias because they do not use individual patient-level data (40). To address this potential bias, the authors used individual patient data from the Flatiron EHR database to create an external RWD-based ceritinib control group using eligibility criteria from the single-arm trials. Additional exclusion criteria were applied to address imbalances between the treatment groups in the stage of diagnosis and crizotinib treatment post-progression. Furthermore, the authors applied a propensity score (estimated based on treatment assignment and prespecified prognostic factors) through an inverse probability treatment weighting to reduce the potential for indication bias (40).

In this trial, OS was compared with a multivariate Cox proportional hazards model (40). As a result of this study, alectinib was associated with a lower risk of death (hazard ratio 0.65; 95% confidence interval [CI] 0.48–0.88) and longer median OS (alectinib: 24.3 months vs. ceritinib: 15.6 months) (40). To support their findings, the authors compared the median survival estimate in the RWD-based ceritinib group with that observed in an independent ceritinib clinical trial cohort (41). The authors concluded that the usefulness of RWD, as a source for constructing external control groups should be evaluated further with more case studies and validated with individual patient data from the control arm from an RCT. As shown by the next examples, other investigators have subsequently explored this topic.

### Case 2: Blinatumomab Compared With Historical Standard Therapy in Adult Relapsed/Refractory Acute Lymphoblastic Leukemia

Blinatumomab (Blincyto) was approved by the FDA in December 2014 and by the European Medicines Agency in November 2015 for the treatment of patients with Philadelphia chromosome-negative relapsed or refractory B-cell precursor acute lymphoblastic leukemia (24, 42). Accelerated approval was based on the results of a phase II single-arm study (MT103-211) in 189 adult patients (42, 43). As a means of providing context for the single-arm study results, the outcomes were compared with those of an external comparator treated with standard-of-care salvage chemotherapy (23, 24).

The historical data set was derived from individual sites in Europe and the United States, along with European national study groups (23). Patients were selected based on key inclusion/exclusion criteria from the single-arm trial. Two analytical approaches were used. In the first approach, estimates of complete remission (CR) and OS in the external comparator group were weighted according to the frequency distribution of predetermined baseline prognostic factors in the single-arm trial population (23). In the second approach, propensity score methods were used to allow for baseline factors to be better balanced between the external control group and blinatumomab-treated patients, and also to enable the quantification of the differences in outcomes between the groups (23).

Both analyses showed favorable results when comparing CR and OS with blinatumomab vs. the benchmark (23). Of note, a phase III RCT subsequently confirmed that blinatumomab treatment was associated with significant improvements in OS and CR compared with chemotherapy (44).

## Use of External Controls Based on RWD in Early-Stage Oncology Drug Development: What Are the Potential Benefits and Challenges?

It has been suggested that for external control groups to gain wider acceptance and use, they will need to demonstrate credibility through repeated use in guiding sponsor decisions of whether to continue or stop the development of drug candidates (5). Following phase Ia dose-escalation and phase Ib dose-expansion studies, sponsors need to decide whether the clinical data warrant advancing development candidates to phase II/III studies. Since phase Ia and Ib studies are typically single-arm studies, external control data could be used to contextualize results and support scientific decision-making. For example, when designing a single-arm phase Ib expansion study in a specific population, an external control group could be constructed from RWD in the same population to create a real-world benchmark and to provide objective thresholds for deciding whether to advance or stop the development of drug candidates based on the eventual study result observed. In this case, characteristics of the populations from the phase I study experimental treatment group and the RWD external control group are aligned as closely as possible, but no direct comparison is necessarily made (i.e., data from each arm are analyzed separately).

In the context of early phase testing of combination therapies, RWD could be used to form single-agent external control groups against which novel drug combinations could be tested and compared. In this way, RWD-based external control groups would be helpful in assessing the contribution of components in combination regimens. These data would also create the opportunity to augment data collected in pivotal studies of novel drug combinations by potentially replacing single-agent arms in phase III studies, when appropriate (45). Furthermore, as described above, real-world datasets may be more likely than RCT data to cover rare diseases or molecularly defined subgroups, and hence may be particularly useful sources of external data for early-stage investigations in these areas.

In a proof-of-concept study published in 2019 (10), Carrigan et al. aimed to assess how closely contemporaneous external control arms constructed from RWD reflected OS observed in control arms from RCTs in advanced NSCLC. The researchers selected patients from the Flatiron Health EHR database who received the same standard-of-care treatment as patients from eight RCTs, and then applied trial-specific eligibility criteria to the EHR dataset.

Propensity score methods were used to achieve greater balance between the groups in terms of baseline covariates. The primary outcome was OS, defined as time from randomization (RCT patients) or treatment initiation (EHR patients) to death. In 10 of 11 analyses conducted, hazard ratio estimates for OS using the external control groups were similar to those from the corresponding RCTs. The study authors suggested that “this is the first study to examine the use of real-world [external control] arms across a number of RCTs using patient-level data to evaluate efficacy directly” (10). The authors concluded that early phase, single-arm oncology trials could be put into context using properly selected control arms derived from contemporaneous EHR data.

## Conclusions

There is growing interest in the use of RWD to complement data from clinical trials, and regulatory authorities such as the FDA, EMA and PMDA have signaled their support for the use of RWD in generating clinical evidence. As noted in its RWE framework, the FDA is considering developing guidance on the use of RWD to construct external control arms to support regulatory decision-making (16). This guidance might provide considerations on when and how to use external control arms and offer insights on specific considerations in clinical development.

Curated EHR datasets may be sufficiently large and detailed to create contemporaneous external control groups (10). With appropriate attention to study design and minimizing bias, an external comparator can provide a benchmark to support interpretation of single-arm study results. Although not currently intended to replace RCTs, the use of external controls based on RWD has the potential to help sponsors make better decisions during early oncology drug development. Ultimately, this could make clinical programs faster and more efficient, and facilitate patient access to important treatments. Maximizing the potential of RWD will require further development of methodologies and acceptance of the data by researchers, healthcare professionals, patients, and regulatory authorities. In the future, the creation of a large centralized cancer patient database or different orthogonal patient databases of curated RWD, which are readily available to be included as a control arm(s), may potentially help to expedite the assessment of RCTs and single-arm trials. However, the set-up of such databases is complex and will require close collaborative approaches between multiple cancer centers, industry partners, patient advocacy groups, regulatory authorities, and others.

## Author Contributions

All authors drafted and/or critically revised the work for important intellectual content, read and gave final approval of the submitted manuscript, were involved in the decision to submit the manuscript for publication, and accept accountability for all aspects of the work.

## Funding

This review was sponsored by Pfizer.

## Conflict of Interest

TAY has received research support (paid to his institution) from Artios Pharma, AstraZeneca, Bayer, Beigene, BioNTech, Bristol Myers Squibb, Clovis Oncology, Constellation Pharmaceuticals, Cyteir Therapeutics, Eli Lilly and Company, EMD Serono, Forbius, F-Star, Genentech, GlaxoSmithKline, Haihe, ImmuneSensor Therapeutics, Ionis, Ipsen, Jounce Therapeutics, Karyopharm Therapeutics, KSQ, Kyowa Hakko USA, Merck & Co., Novartis, Pfizer, Regeneron, Repare, Ribon Therapeutics Inc, Sanofi, Scholar Rock, Seattle Genetics, Tesaro, and Vertex Pharmaceuticals; and has received fees for consulting from Aduro Inc, Almac, Artios, AstraZeneca, Athena, Atrin Pharmaceuticals, Axiom, Bayer, Bristol Myers Squibb, Calithera Biosciences, Clovis Oncology, Cybrexa Therapeutics, EMD Serono, F-Star, GLG, Guidepoint, Ignyta, I-Mab Biopharma, ImmuneSensor, Janssen, Merck & Co., Pfizer, Repare Therapeutics, Roche, Schrodinger, Seattle Genetics, Varian Medical Systems, Zai Lab and ZielBio. LA has received personal fees from Janssen and Pfizer. IJ and LJL are full-time employees of and hold stock/options in Pfizer. DB was a full-time employee of and held stock/options in Pfizer when the manuscript was in development. EB is an employee of Roche Diagnostics but was a full-time employee of Pfizer when the manuscript was in development and holds stock/options in Pfizer and Roche.

The authors declare that this study received funding from Pfizer. The funder had the following involvement with the study: provided funding for medical writing and editorial support.

The reviewer JL declared a past co-authorship one of the authors TY to the handling Editor.

## Publisher’s Note

All claims expressed in this article are solely those of the authors and do not necessarily represent those of their affiliated organizations, or those of the publisher, the editors and the reviewers. Any product that may be evaluated in this article, or claim that may be made by its manufacturer, is not guaranteed or endorsed by the publisher.
